# Multi-parametric cell profiling with a CMOS quad-modality cellular interfacing array for label-free fully automated drug screening

**DOI:** 10.1039/c8lc00156a

**Published:** 2018-09-26

**Authors:** Jong Seok Park, Sandra I. Grijalva, Moez K. Aziz, Taiyun Chi, Sensen Li, Michael N. Sayegh, Adam Wang, Hee Cheol Cho, Hua Wang

**Affiliations:** a The School of Electrical and Computer Engineering, Georgia Institute of Technology, Atlanta, GA 30308, USA.; b The Department of Biomedical Engineering, Emory University, Atlanta, GA 30332, USA

## Abstract

Cells are complex systems with concurrent multi-physical responses, and cell physiological signals are often encoded with spatiotemporal dynamics and further coupled with multiple cellular activities. However, most existing electronic sensors are only single-modality and cannot capture multi-parametric cellular responses. In this paper, a 1024-pixel CMOS quad-modality cellular interfacing array that enables multi-parametric cell profiling for drug development is presented. The quad-modality CMOS array features cellular impedance characterization, optical detection, extracellular potential recording, and biphasic current stimulation. The fibroblast transparency and surface adhesion are jointly monitored by cellular impedance and optical sensing modalities for comprehensive cell growth evaluation. Simultaneous current stimulation and opto-mechanical monitoring based on cardiomyocytes are demonstrated without any stimulation/sensing dead-zone. Furthermore, drug dose-dependent multi-parametric feature extractions in cardiomyocytes from their extracellular potentials and opto-mechanical signals are presented. The CMOS array demonstrates great potential for fully automated drug screening and drug safety assessments, which may substantially reduce the drug screening time and cost in future new drug development.

## Introduction

Cells are the basic structural and functional building blocks of living organisms. Understanding complex cell physiology, such as cell function, proliferation, apoptosis, and morphology, is a fundamental step to advance biological scientific research and bio-technology development.^1–6^

Cell-based assays employ living cells as “sensor front-ends” to perform label-free biochemical sensing through cellular physiology that converts biochemical signals to easily measurable physical signals by underlying electronic sensors.^7–16^ Compared to traditional biochemical assays or electronics-only sensors, cell-based assays provide physiologically relevant information and offer accurate representations of real-life models. Moreover, they can achieve high sensitivity, selectivity, and fast sample-in-answer-out response time, which can be potentially improved by further genetic engineering the “front-end” cells. In practice, cell-based assays are widely used in the pharmaceutical industry to screen and down-select drug leads or test new drugs for efficacy, pharmacodynamics, pharmacokinetics, and cytotoxicity.

Most existing cell-based assays utilize single-modality electronic sensors, each of which only measures one cell physiological property. Examples include microelectrode arrays (MEAs) with extracellular potential amplifiers and stimulators for recording neuron activities and action potential conduction,^17–21^ electrical impedance sensors for cell-growth assay, cardiac beating measurements, and myocardial ischemia detection,^22–24^ ion-sensitive field effect transistor (ISFET) arrays for cell metabolism assay,^25,26^ magnetic sensor arrays for capturing cardiac beating or molecular detection,^12,16,27–29^ and optical sensors for DNA sensing and sequencing.^30–34^

However, cells are highly complex systems with concurrent multi-physical activities, and thereby holistic understanding of such complex cellular physiological responses remains a daunting task. For example, cells perform various metabolic activities such as anabolism and catabolism, exhibit electrical activities with excitable membranes, and experience mechanical activities such as chemotaxis, phototaxis, and geotaxis. Also, cells communicate with each other through a wide variety of chemical and physical signals. Therefore, real-time and multi-parametric cell profiling and modelling are of paramount importance to enable decoding and decoupling complex cellular signals and identify target pathways for comprehensive understanding of cell physiology.

Multi-parametric cell profiling and modelling are particularly critical for drug screening and drug safety/toxicity assessment.^35–37^ In the early stages of drug development before clinical trials, phenotypic drug screening is often performed, so that large numbers of drug compounds are screened using many panels of disease-relevant cell lines to identify target pathways and potential drug candidates. Furthermore, extensive cell-based assays are utilized to assess the efficacy and toxicity of the selected compounds. The drug development cost increases exponentially at the later stages of new drug development largely due to expensive and low throughput animal testing and clinical trials.^38,39^ It has been recently reported that due to late stage failures, the return on investment (ROI) for pharmaceutical companies may drop to as low as 5%.^40^ Moreover, the cost of drug withdrawal from the market due to drug-induced organ toxicity is even higher.^41–43^ For example, terfenadine was withdrawn from the market due to cardiac toxicity with an estimated loss of US $6 billion.^42^ Therefore, it is essential to down-select failure drug leads and identify drug-induced problems at the early stage.^38,39^ As a result, it is indispensable to use cost-effective cell-based assays to investigate the cytotoxic effects of drug compounds with a wide variety of healthy and disease-relevant cells, so that expensive animal testing and clinical trials are only used on the most promising drug leads. However, due to the massive combination of compound libraries and disease-relevant cell lines, as well as extensive drug cytotoxicity test sets, *i.e*., 5–6 million tests,^8^ phenotypic drug screenings are often extremely time-consuming and labour-intensive. In parallel, kinetic and multi-parametric cell profiling is essential to capture unknown drug effects or real-time cellular behaviours, which exacerbates the complexity and labour cost of phenotypic drug screening using conventional single-modality sensors. Thereby, a fully automated and low-cost drug screening platform supporting real-time multi-parametric cell profiling is necessary, so that the cost and time for new drug development can be substantially reduced.

**Fig. 1:**
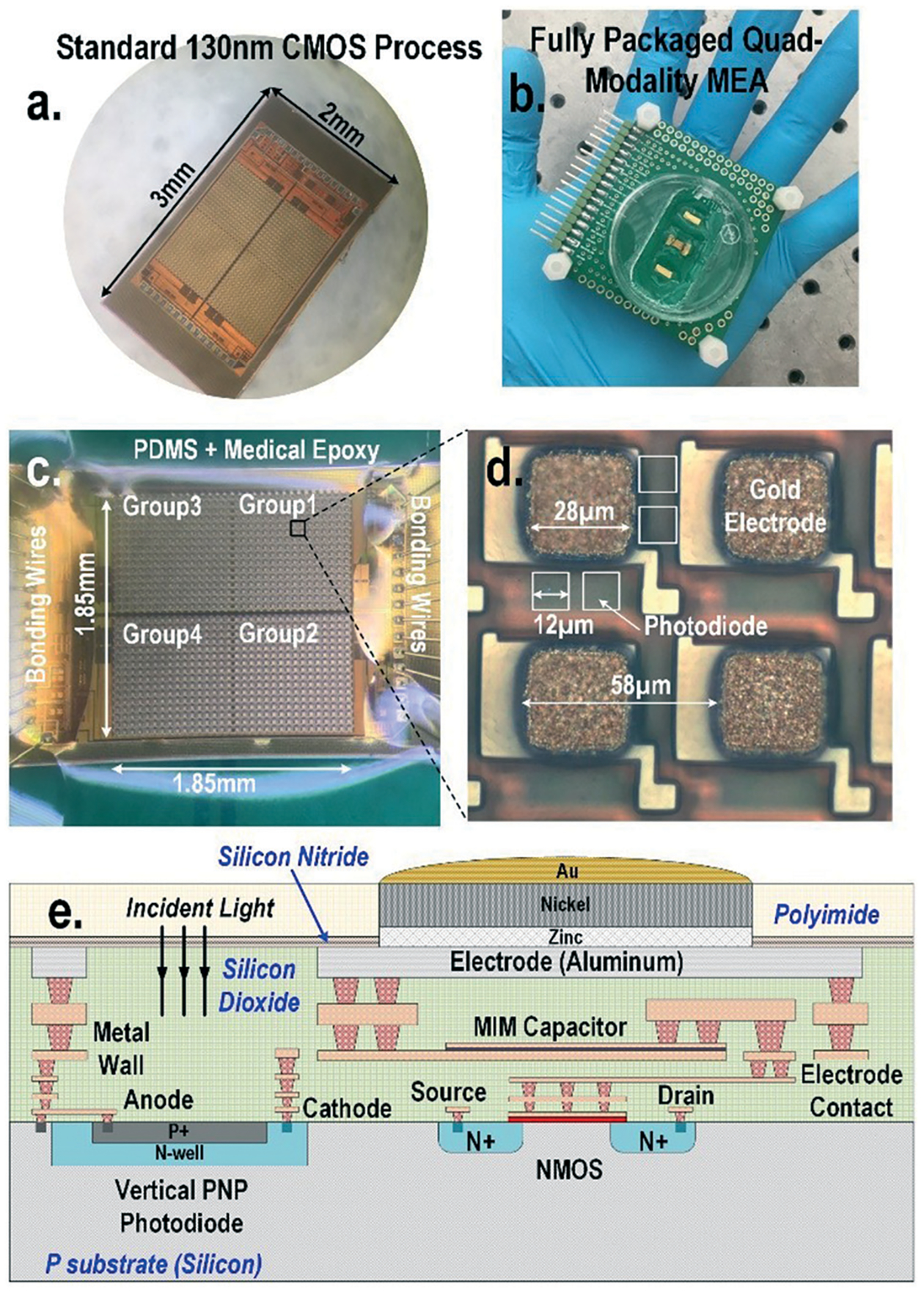
(a) CMOS quad-modality cellular interfacing array. The device features extracellular potential recording, cellular impedance measurement, static and dynamic optical recordings, and biphasic current stimulation. (b) Fully packaged quad-modality array chip. (c) The zoomed-in view of the packaged array. The chip is encapsulated by biocompatible materials such as PDMS and medical epoxy with the sensor area exposed for cell culture and cellular interfacing. (d) Each multi-modality pixel contains one 28 μm × 28 μm electrode and four 12 μm ×12 μm photodiodes and in-pixel front-end circuitry. (e) The side view of CMOS quad-modality cellular interfacing array.

To address these challenges, in this paper, a 1024-pixel CMOS quad-modality cellular interfacing array to enable real-time multi-parametric cell profiling is presented (Fig. 1). The proposed chip features multi-modality cellular sensing and actuation within each pixel, including 1) cellular impedance sensing, 2) static and dynamic optical recordings, 3) extracellular potential recording, and 4) *in situ* biphasic current stimulation. The detailed operations of the CMOS chip, its electrical characterization, and the biocompatible packaging techniques are explained in references.^44,45^ Fibroblasts and neonatal rat ventricular myocytes (NRVM) are successfully cultured on-chip. The time-lapse cell transparency/density and cell-to-surface adhesion of the on-chip cultured fibroblasts are measured by two distinct sensing modalities: static optical opacity detection and cellular impedance characterization. This joint two-modality measurement comprehensively monitors cell migration, proliferation, and viability. Additional time-lapse fibroblast detachment assays with trypsin enzyme are performed for further verification. In addition, simultaneous biphasic current stimulation and optical response monitoring based on cardiomyocytes are demonstrated without any monitoring dead-zone area caused by electrical stimulation artefacts. The real-time optical transparency modulation of on-chip cultured cardiomyocytes is due to their cardiac muscle contraction and relaxation events and is readily measured by in-pixel photodiodes, enabling cardiomyocyte stimulation and response monitoring at the same time and location without any monitoring dead-zone. Furthermore, this real-time optical transparency modulation reveals critical physiological parameters related to the mechanical beating of cardiomyocytes. The multi-parametric isoproterenol dose-dependent feature extractions based on extracellular potential signals and optical signals are also presented with a statistical summary.

## Materials and methods

### A CMOS quad-modality sensor/stimulator cellular interfacing array

The quad-modality CMOS joint sensor/simulator array features extracellular potential recording, static and dynamic optical recordings, cellular impedance sensing, and biphasic current stimulation for on-chip cultured cell samples (Fig. 1). The array consists of 4-pixel groups each with 256 multi-modality pixels at a pixel–pixel pitch of 58 μm, and every individual pixel can be arbitrarily accessed. Each multi-modality pixel comprises one 28 μm × 28 μm gold-plated electrode and four 12 μm × 12 μm photodiodes, and thus each array chip achieves a total of 1024 electrical sensing/stimulation sites and 4096 optical recording sites with a 1.85 mm × 1.85 mm tissue-level field-of-view (FoV) per chip. The CMOS array chip occupies 2 mm × 3 mm including all the 1024 multi-modality pixels, pixel-group circuits, input/output (I/O) pads, and other auxiliary on-chip circuits. The detected cellular signals are processed by on-chip signal-conditioning blocks. The quad-modality array is fully programmable through the on-chip serial-to-parallel interface (SPI), while the 4 parallel output signals corresponding to 4-pixel groups are read out by the FPGA (Measurement Computing USB-1616HS).

The operations of each modality are briefly explained as follows. For extracellular potential recording, two electrodes, one for sensing and the other for reference, are selected from two arbitrary pixels and a differential amplifier is configured for these two electrodes to enable differential extracellular potential recording with a large suppression (>60 dB) of common-mode noise or perturbations. For cellular impedance sensing, the electrodes of the two adjacent pixels are selected; one for voltage excitation and the other for current termination, and the cellular impedance including the cell-surface contact impedance in the vicinity of the two electrodes is measured. Since two adjacent electrodes with a fixed pixel pitch size of 58 μm are selected, there is no geometry-dependent or location-dependent impedance variations.^21^ The selected two electrodes, *i.e*., vertically adjacent electrode pairs or horizontally adjacent electrode pairs, are scanned through the entire CMOS chip to achieve complete 2-D cellular impedance characterization. For static optical imaging, the entire 4096 optical recording sites are scanned through the CMOS chip, while for dynamic optical recording, one optical recording site per pixel group is selected, and the real-time received light intensity is measured. For the cellular stimulation, two arbitrary electrodes in the same pixel group are selected and connected to the current stimulator that sends fully programmable charge balancing biphasic current pulses.

The CMOS chip supply voltage is 3 V and the peak DC power consumption for optical sensing, impedance detection, cellular potential recording, and stimulation are 84 mW, 30 mW, 6 mW, and 9 mW, respectively. The measurement time to scan the entire sensing area (1.85 × 1.85 mm^2^) is 1.18 seconds and 9.6 seconds for optical and impedance sensing, respectively.

### Electrode modification and biocompatible packaging

The diced CMOS chip is packaged with biocompatible materials (Fig. 1). The native material for the CMOS electrodes and input/output pads is aluminium from the manufacturer. Since aluminium dissolves easily in cell culture medium and the resulting aluminium ions can incur severe cellular toxicity, aluminium electrodes must be protected and well covered by a noble metal coating such as gold and platinum. This is particularly important when the electrodes are also used for current-based cell stimulation. The composite metal layer of Zn/Ni/Au is deposited on top of the aluminium electrode for reliable electro-chemical protection by following an electro-less gold plating procedure. First, the diced CMOS chips are immobilized onto a glass substrate using polydimethylsiloxane (PDMS) for handling. The CMOS chips are sequentially washed by acetone, methanol, and isopropyl alcohol, and immersed into the aluminium etchant (TRANSENE) to remove the aluminium oxide layer. Next, the glass substrate with the CMOS chips is sequentially immersed into Zincate (TRANSENE), Nickelex (TRANSENE), and immersion gold solution (TRANSENE) to deposit a thin gold layer of ~200 nm. Finally, the chips are immersed in autocatalytic gold solution (UYEMURA) to grow the gold layer up to 1 μm *via* autocatalytic gold plating from the immersion gold layer as the seedling layer in this process.

The gold-plated CMOS chips are then removed from the glass substrate and permanently attached to a supporting 5 cm × 5 cm FR4 printed circuit board (PCB). The I/O pads of the chip are wire-bonded to the PCB traces using gold bonding wires with a diameter of 28 μm. The bonding wires are first encapsulated in medical epoxy (EPOTEK) for electrical isolation, while the pixel area is exposed without epoxy to allow for on-chip cell culture and contact-based cellular sensing. Then, an additional PDMS layer is deposited on the cured medical epoxy for improved biocompatibility. Finally, a standard 35 mm cell culture plate with a laser-cut bottom is mounted on the PCB to hold the cell samples and culture medium.

### Neonatal rat ventricular myocyte and fibroblast on-chip culture

Neonatal rat ventricular myocytes (NRVMs) and cardiac fibroblasts are isolated from 1 to 2 day-old neonatal rats and cultured as monolayers as previously described.^45,71–73^ NRVMs are transduced with an Ad-GFP vector (MOI = 1–2) for 2 hours in suspension at room temperature (RT) and then seeded overnight on the CMOS chip. NRVM spheroids are formed using AggreWellTM (STEMCELL, Vancouver, Canada). A total of 0.5 mL of rinsing solution (STEMCELL, Vancouver, Canada) is added to each well and the plate is centrifuged for 5 min at 2000 × *g*. The plate is washed with cell culture medium prior to use, and 0.5 mL of medium is added to the sample well and centrifuged for 3 min at 2000 × *g* to remove bubbles. Cell samples containing 1.2 × 106 NRVMs in a 0.5 mL volume is added to each well. The AggreWell plate is centrifuged for 5 min at 200 × *g*. The plate is incubated overnight, and its medium is replenished by removing 0.5 mL from the edge of the well and adding 0.5 mL fresh medium into the well. After three nights, the spheroids have formed and can be recovered from the plate by gently jetting them out with a pipette.

Cardiac fibroblasts are formed using a 12-well Corning plate (Corning, Corning, NY) coated with 2 mL of 1% autoclaved agarose gel. Their hydrophobic coating allows the cardiac fibroblasts to spontaneously form spheroids of different sizes ranging from 30–500 μm. Cardiac fibroblasts are maintained in DMEM supplemented with 10% FBS (GE Healthcare, Pittsburgh, PA), 2 mM GlutaMAX, penicillin–streptomycin, and MEM non-essential amino acids (ThermoFisher Scientific). The cardiac fibroblasts are also stained for fluorescence imaging using calcein-AM (ThermoFisher Scientific). Trypsin–EDTA 0.05% (ThermoFisher Scientific) was used to detach the spheroids. The cardiomyocyte spheroids and fibroblast aggregates are pipetted directly onto the CMOS chip. Experiments with murine subjects were performed in compliance with the relevant laws and institutional guidelines and were approved by the IACUC of Emory University.

### Measurement set-up

The I/O of the quad-modality CMOS cellular interfacing array are accessed through pin headers in the PCB. The digital control codes are generated by the field-programmable gate array (Measurement Computing USB 1616HS) and streamed into the chip through the on-chip SPI at a clock frequency of 2 MHz. The cellular output signals of the CMOS chips are acquired by the FPGA board and processed in MATLAB. The chip power can be supported by D-type batteries to minimize the 60 Hz power line noise, and the electrical and cell-based measurements are performed in a custom-designed Faraday metal cage for proper electromagnetic shielding.

## Results and discussion

### Multi-modality label-free time-lapse cell transparency and cell-to-surface adhesion measurements of on-chip cultured fibroblasts

Cell-to-surface adhesion and cell transparency are critical phenotypic parameters for functional drug screenings and drug toxicity/safety assessments.^11,23,42,46–51^ Cell-to-cell and cell-to-extracellular-matrix (ECM) adhesive properties are closely related with important physiological processes, such as tissue organization, cell viability and proliferation,^49^ and cell migration.^50^ Moreover, cell adhesion assays are essential to investigate the activation of membrane-bound proteins and interactions with extracellular microenvironments,^51^ which play central roles in disease pathogenesis, such as cardiac fibrosis and cancer metastasis, wound healing, and therapeutic target identification. In parallel, optical opacity offers an additional and orthogonal modality for monitoring cell viability, proliferation, and migration, while providing unique information about cell morphology, density, and contractile function.^52,53^ While immunohistochemistry and microscopy are routinely used to assay cell viability, proliferation, and migration, the required labelling procedures and complex imaging set-ups preclude long-term, repeated, and kinetic observation and manipulation, especially at the scale required for high-throughput drug testing platforms. Furthermore, repeated application of fluorescent stains could result in photo-toxicity and cell damage. In this section, time-lapse cellular impedance images and optical opacity images based on the same on-chip cultured fibroblast sample are demonstrated to track cell density and surface adhesion for holistic cell growth assay.

**Fig. 2:**
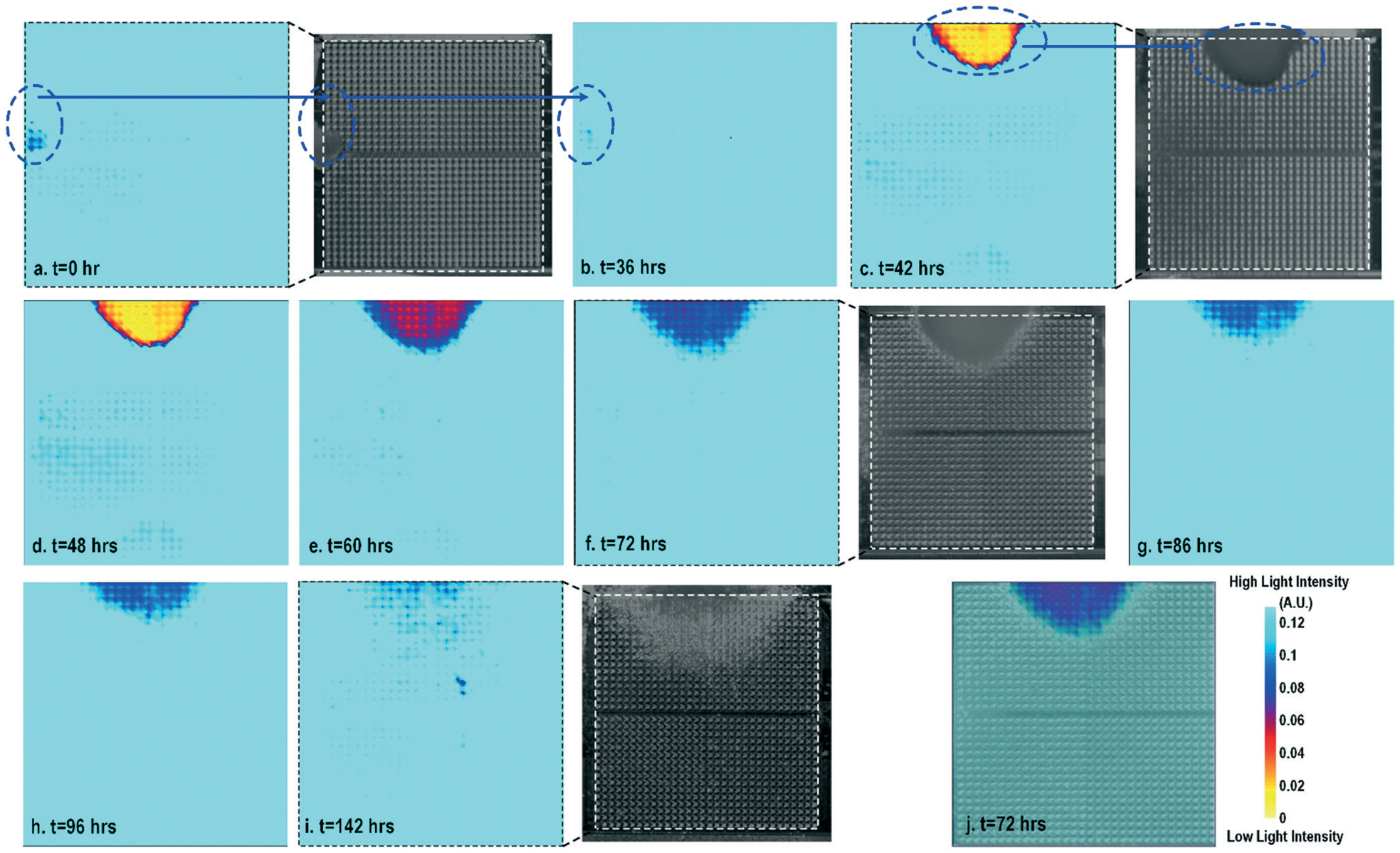
(a)–(i) Measured time-lapse optical opacity images of on-chip cultured fibroblasts for cell growth assays together with the corresponding reference stereo-microscope images. (j) The reference stereo-microscope image and the CMOS optical opacity image at *t* = 72 h are superimposed to show on-chip fibroblast location and matching. All optical opacity images use the same scale as that in (j).

Fig. 2 shows the measured time-lapse optical opacity images of the on-chip cultured fibroblasts together with standard stereo-microscope images as the reference. In this experiment, a fibroblast aggregate is first pipetted on the left side of the quad-modality array at *t* = 0 (the dotted line in Fig. 2a) and later a larger fibroblast aggregate is pipetted on the top side of the array at *t* = 39 h (the dotted line in Fig. 2c). Fibroblasts are principal active cells of connecting tissues and often pre-cultured to deposit ECM. Fibroblasts proliferate and secrete a gel-like abundant matrix, creating enriched microenvironments.^54^ The time-lapse stereo-microscope images (Fig. 2) show a gradual opacity decrease for 142 hours, implying that the fibroblasts have successfully attached and migrated on the chip. Similar changes are observed by the optical sensing modality of our CMOS quad-modality cellular interface array. The time-lapse measured optical opacity images show the 2-dimensional orthographic projection of fibroblasts onto the entire CMOS chip active sensing area (1.85 × 1.85 mm^2^). The measured opacity images in Fig. 2 clearly show the location, boundaries, and internal opacity gradients of the fibroblast aggregate seeded onto the array. The fibroblast aggregate pipetted at the left side of the CMOS chip (*t* = 0 h) is captured in the measured optical opacity image in Fig. 2a. Then the opacity of the fibroblast decreases due to the cell migration and reduced local cell density at *t* = 36 h (Fig. 2b). Similar behaviours are observed in the fibroblast aggregate pipetted at the top side of the CMOS chip at *t* = 39 h (Fig. 2c–i). The fibroblast aggregate boundary first expands in its radial direction due to the cell proliferation and then fades out due to cell migration away from the centre of the cluster (Fig. 2c–i). The reference stereo-microscope image and measured optical opacity image at *t* = 72 h are superimposed in Fig. 2j, showing excellent matching. The fibroblast aggregates achieve homogeneous quasi-transparency at *t* = 142 h, closely matching the microscope imaging and implying successful cell proliferation and migration (Fig. 2i). At a later culture stage, optical detection is less effective to monitor fibroblasts due to decreased cell opacity/density caused by migration. Moreover, optical sensing alone cannot successfully discriminate cell viability, attachment, and function, since floating dead cells and healthy transparent cells cannot be distinguished by opacity-only measurements.

**Fig. 3:**
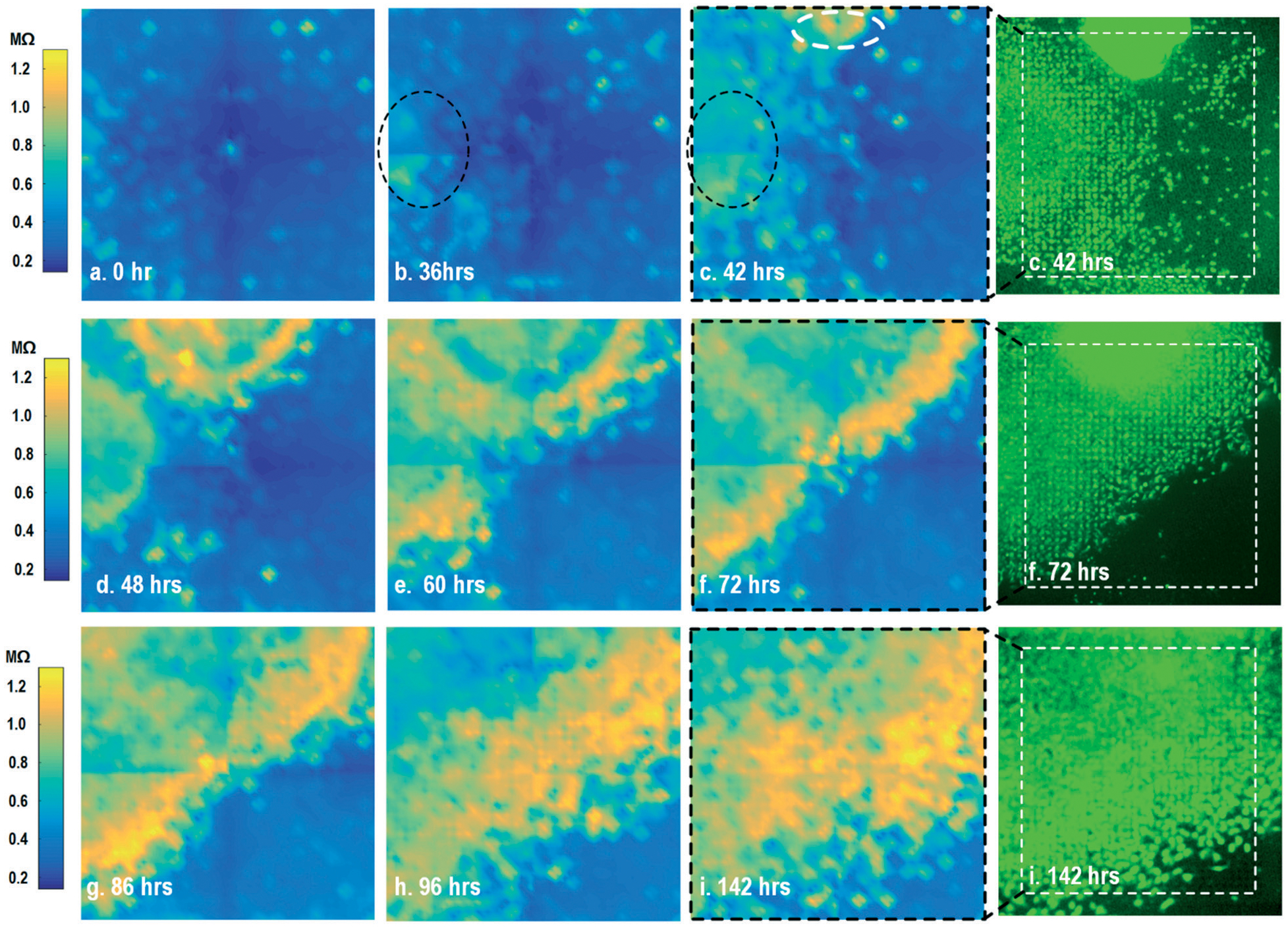
Measured time-lapse cellular impedance images of on-chip cultured fibroblasts together with reference fluorescence images. The cellular impedance measurement is performed at 100 kHz and the impedance magnitude is plotted.

By leveraging the quad-modality operation of our CMOS array, cellular impedance sensing in conjunction with optical opacity sensing is performed on the same cellular sample to characterize the cell-to-surface adhesion. Therefore, cellular opacity/density and attachment can be comprehensively monitored with the two joint modalities. Fig. 3 shows the time-lapse impedance magnitude images of the on-chip cultured fibroblasts together with the reference fluorescence microscope images. The cellular electrical impedance is measured at 100 kHz to yield accurate extracellular information.^46^ Note that the cellular impedance measurements (Fig. 3) are performed in conjunction with optical measurements (Fig. 2) on the same cellular sample, ensuring good temporal and biological correlations of the two measurements. The time-lapse cellular impedance images track the advancing front of fibroblasts in the radial direction with increasing cell adhesion to the chip surface. The fibroblast aggregate pipetted on the top side of the chip starts to appear in the impedance image in Fig. 3c. Then, it expands and enhances its surface adhesion over time (Fig. 3c and d). Eventually, the two fibroblast aggregates, separately pipetted at *t* = 0 and *t* = 39 h, merge together (Fig. 3e) and further expand on the CMOS chip surface (Fig. 3f–h), achieving an almost full confluency at *t* = 142 h (Fig. 3i), as confirmed by the reference fluorescence image. The impedance image in Fig. 3c is measured 3 h (*t* = 42 h) after the fibroblast aggregate is pipetted on the top side of the chip. By comparing it to the parallel measured optical opacity image at *t* = 42 h (Fig. 2c), the measured impedance image (Fig. 3c) reveals that within 3 h, only a small part of the fibroblast aggregate (white dotted) achieves adhesion to the chip surface. Then, this surface adhesion gradually expands over time (Fig. 3d–i), which however cannot be easily visualized by means of optical opacity images (Fig. 2). For the reference fluorescence images, a calcein AM green-fluorescent marker (ThermoFisher Scientific) is added to the on-chip cultured fibroblasts to indicate cell viability and distribution. The reference fluorescence images closely match the corresponding cellular impedance images (Fig. 3c, f and i). Therefore, for time-lapse cell growth assay, our CMOS quad-modality array may potentially augment or even replace expensive, bulky, and low throughput fluorescence imaging systems, resulting in significant cost reduction, throughput increase, and long-term cellular monitoring.

**Fig. 4:**
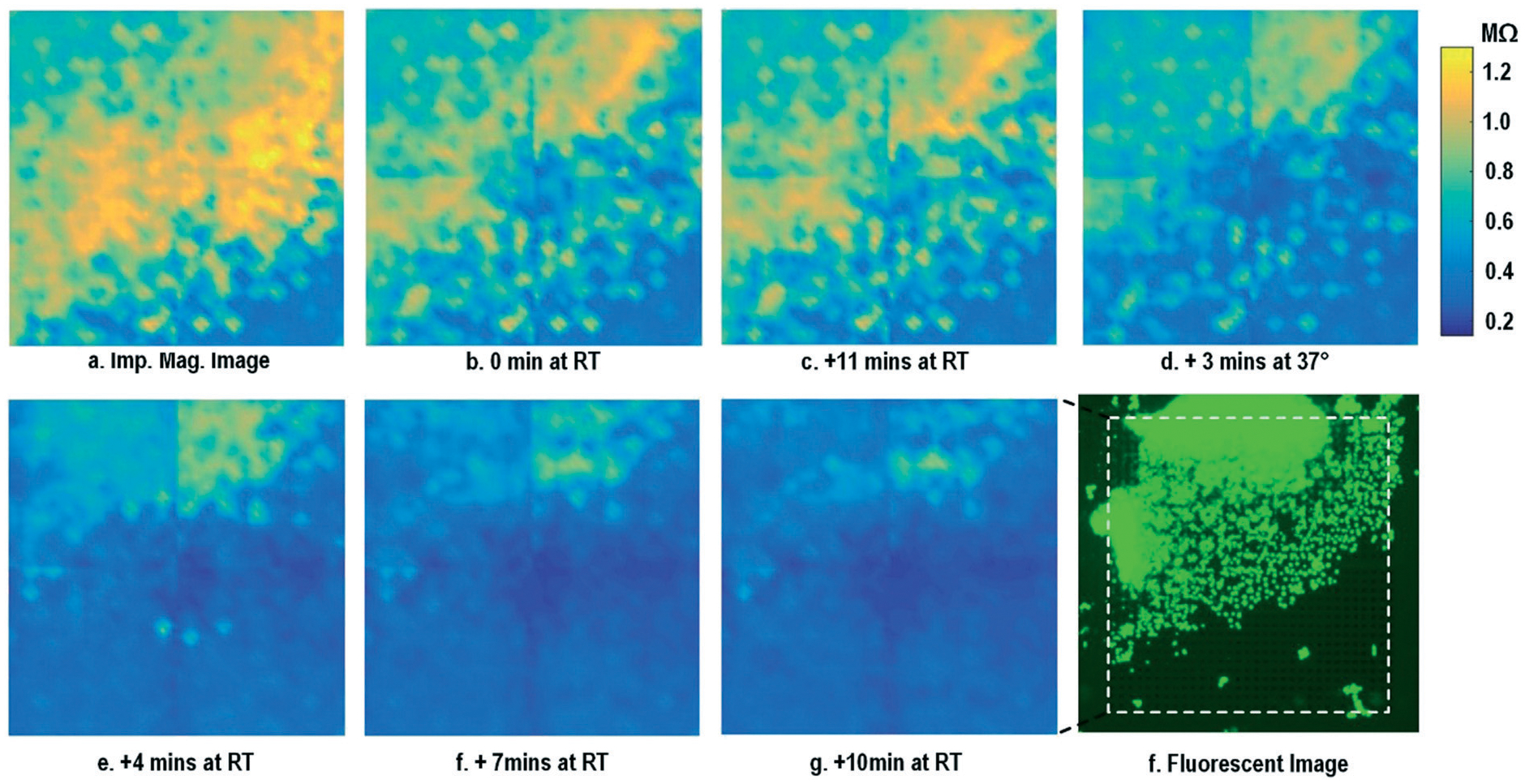
Measured time-lapse impedance magnitude images of on-chip cultured fibroblasts after trypsin enzyme administration. Note that Fig. 4a corresponds to Fig. 3i. The cellular impedance measurement is performed at 100 kHz and the impedance magnitude is plotted.

After the on-chip cultured fibroblasts achieve an almost full confluency (Fig. 3i), cell detachment assay is performed to further verify the cell adhesion measurements. Trypsin enzyme (ThermoFisher Scientific) is added to the on-chip cultured fibroblasts (Fig. 4a). Trypsin is an enzyme widely used to detach adherent cells from their substrates and initiates cell–cell dissociation. After trypsin administration, time-lapse cellular impedances are measured and shown in Fig. 4. The measured cellular impedance (Fig. 4b) shows that the large part of fibroblasts is detached with trypsin administration. However, a slow cell detachment process is observed at RT and stops at *t* = 11 min, as shown in Fig. 4b and c, since the trypsin optimal activity temperature is 37 °C and not RT. Then, the cell-loaded CMOS chip is incubated at 37 °C, and the impedance images show a significantly increased cellular detachment after 3 min incubation (Fig. 4d). A final impedance measurement is taken at 21 min after moving the CMOS chip from the incubator (Fig. 4g), clearly showing a lower cellular impedance and thus indicating cell detachment. In comparison, a corresponding fluorescence microscope image (Fig. 4f) is shown at *t* = 21 min, which, however, only captures the 2D projection of the cellular image and cannot reveal cell-surface attachment. With mechanical agitation, the cells are observed to be indeed detached and are floating in the medium using a fluorescence microscope. These cell detachment experiments further verify that the impedance measurement can successfully monitor the cell-to-surface adhesion, while optical imaging alone cannot.

**Fig. 5:**
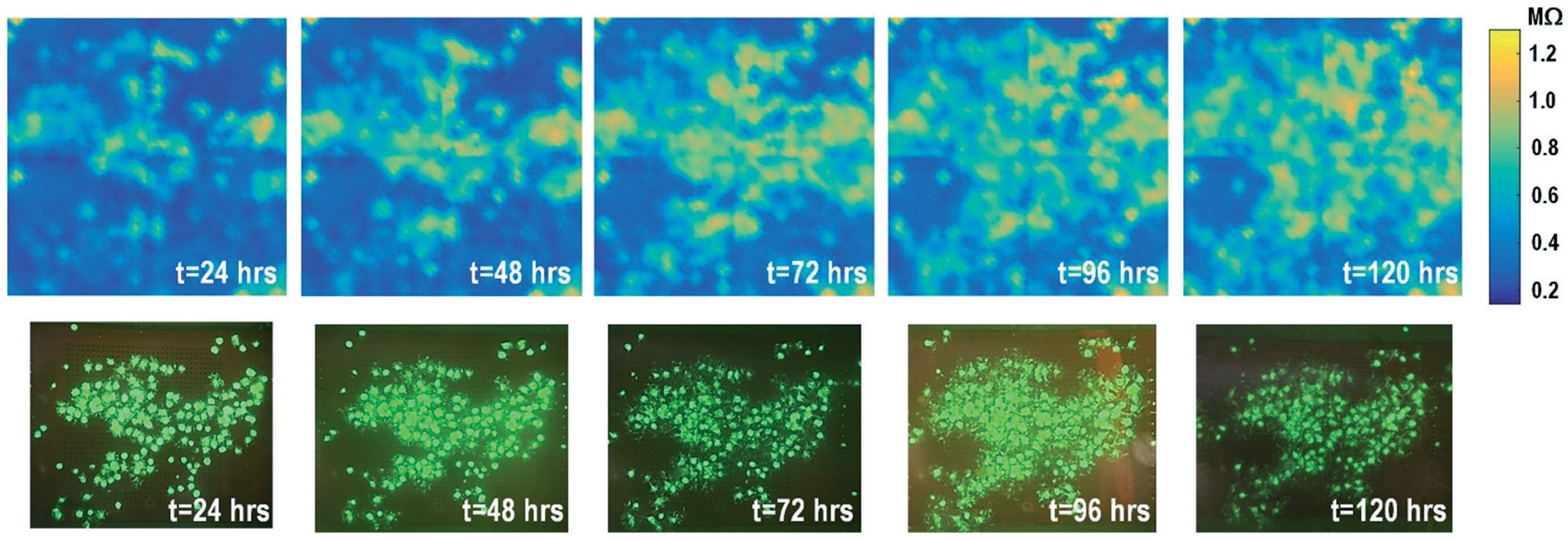
Measured time-lapse impedance images of on-chip cultured rat cardiomyocyte spheroids together with reference fluorescence images. The cellular impedance measurement is performed at 100 kHz and the impedance magnitude is plotted.

The time-lapse impedance measurements are also performed using rat cardiomyocyte spheroids. The measured time-lapse impedance images are shown in Fig. 5 and match well with the reference fluorescence microscope images. The measured time-lapse impedance images show that the on-chip cultured cardiomyocyte spheroids are slightly spreading and their surface attachment is enhanced over time (96 h). The measured impedances of the on-chip cultured cardiomyocyte spheroids are between 0.5 MΩ and 0.8 MΩ, which are lower than the impedance of the on-chip cultured fibroblasts of around 1–1.2 MΩ. This matches well with the understanding that fibroblasts typically show strong surface adherence due to their proliferation and extracellular matrix secretion.^54^

### Simultaneous electrical current stimulation and cellular response monitoring using dynamic label-free optical sensing

For electrically excitable cells, it is critical to perform cellular monitoring simultaneously with electrical excitation to investigate various neurological and cardiovascular diseases.^55–59^ Since electrical stimulation can regulate the firing frequency of excitable cells and precisely control spatiotemporal initiation of the action potential cycles, it is essential in various cardiomyocyte and neuronal studies. Moreover, it has been recently reported that electrical stimulation can promote cardiac differentiation^60^ and maturation of cardiomyocytes.^61^ However, simultaneous electrical stimulations and extracellular potential recordings on the same cellular samples remain a major challenge in practice, since the stimulation artefacts often saturate the extracellular potential recording amplifiers.^62,63^ The required voltage amplitude of electrical stimulation is typically between 0.1 V and 10 V for successful cell pacing, while the evoked extracellular potential amplitude is only around 100 μV.^17–21^ Therefore, simultaneous electrical stimulations and potential recordings require >60 dB real-time broadband stimulation artefact cancellation. In practice, this problem is mitigated by performing extracellular potential recordings at a distant site from the electrical stimulation site, since the stimulation artefact propagating through this spatial distance will be attenuated and delayed, so the artefact can be separated from the evoked extracellular potentials.^19,20^ However, such an arrangement results in a large monitoring dead-zone area of cells around the stimulation location, typically >200 μm × 200 μm,^19^ where extracellular potential recordings cannot be performed and the electrical activity of the cells, thus, cannot be monitored.^19^ On the other hand, successful electrical stimulation of cardiomyocytes will invariably lead to synchronized cell beating and pulsatile mechanical movement;^64,65^ our quad-modality CMOS array is utilized to perform electrical stimulations on the cardiomyocytes and simultaneously record their synchronized mechanical movements by optical sensing. With electrical simulations and optical measurements, this cross-domain operation naturally guarantees signal isolation and allows simultaneous stimulation and recording of the same cardiomyocyte sample at the same location without any monitoring dead-zone.

**Fig. 6:**
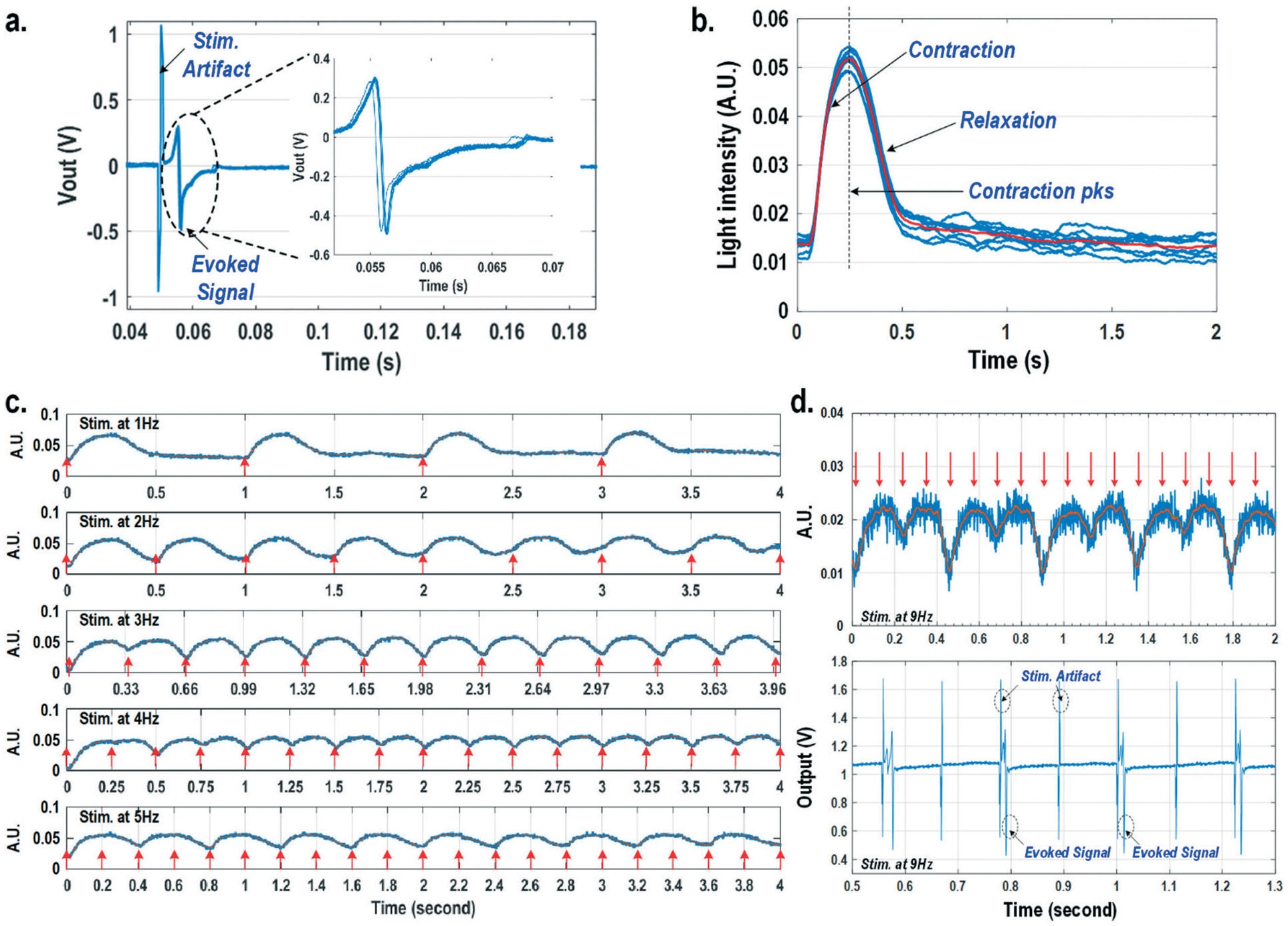
(a) The overlay plot of the measured extracellular potentials with a current stimulation pulse width of 800 μs. (b) The overlay plot of the measured optical signals at a stimulation rate of 0.5 Hz. (c) The measured optical signals at different stimulation rates. (d) The measured optical signals and extracellular potentials at a stimulation rate of 9 Hz.

For this experiment, neonatal rat ventricular myocytes (NRVM) are cultured on a fibronectin coated quad-modality CMOS cellular interface array chip. The stimulation capture rates of on-chip cultured rat cardiomyocytes are first characterized *versus* biphasic current pulse widths at a fixed current amplitude of 8 μA. For the capture rate characterization, extracellular potential recording is enabled at the recording pixel that is 422 μm away from the biphasic current stimulation pixel to ensure sufficient stimulation artefact attenuation and evoked signal response delay. Fig. 6a shows an overlay plot of the measured extracellular potentials for 10 continuous current stimulations. With a pulse width of 800 μs, a current amplitude of 8 μA, and a pulse frequency of 0.5 Hz, 10 evoked signals for 10 continuous current stimulations are observed, achieving a 100% stimulation capture rate. Furthermore, the evoked signal propagation delay after stimulation, spike amplitude, and spike shape are consistent for 10 continuous current stimulations (Fig. 6a).

After the capture rate characterization, simultaneous dynamic optical recording and biphasic current stimulation are demonstrated on a single pixel. For dynamic optical recording, the cell-loaded CMOS cellular interfacing array is placed in a dark box with an off-the-shelf LED light source installed at the top of the dark box, and an LED light with constant intensity emits towards the array. The in-pixel photodiodes below the cardiomyocytes measure the received light intensity in real-time and the real-time intensity variations are due to the transparency modulation associated with cardiomyocyte contraction and relaxation mechanical beating. Fig. 6b shows an overlay plot of the measured real-time received light intensity for 10 continuous beats for the on-chip cultured rat cardiomyocytes with a concurrent biphasic current stimulation at 0.5 Hz, a 1.2 ms pulse width, and a 8 μA stimulation current amplitude. The measured real-time light intensity faithfully captures the cyclic beating patterns of cardiomyocytes upon stimulation at 0.5 Hz. The measured light intensity waveforms (Fig. 6b) consistently show that upon current stimulation, the light intensity increases with time until it reaches a peak light intensity, and then it decays to a resting light intensity. This increased light intensity corresponds to cardiac muscle contraction, and the following decaying light intensity corresponds to cardiac muscle relaxation. The dynamic optical recording captures real-time cardiac muscle opto-mechanical responses, which closely relate to intracellular calcium transients, and thus reveals critical cellular parameters for investigating cardiac arrhythmia.^64^ The measured average time to reach the contraction peak after stimulation is 190.3 ms with a standard deviation of 13.2 ms (Fig. 6b), and the details of the multi-parametric feature extraction will be discussed in the following section.

Next, the stimulation rate is increased up to 5 Hz and the resulting optical signals are shown in Fig. 6c. The stimulation capture rate is kept at 100% up to a 4 Hz stimulation rate and drops to 50% at a 5 Hz stimulation rate with a fixed pulse width of 1.2 ms and a fixed current amplitude of 8 μA. Fig. 6c shows that at a stimulation rate of 3 Hz corresponding to a stimulation period of 333 ms, the subsequent stimulation pulse is applied to cardiomyocytes before they complete the relaxation phase of the contraction cycle. However, cardiomyocytes are still successfully paced indicating that the excitation was applied during the relative refractory period. At a stimulation rate of 4 Hz corresponding to a period of 250 ms, the subsequent stimulation pulse is applied at 59.7 ms after the cardiac muscle reaches the contraction peak (190.3 ms). Although the cardiac muscle relaxation period is significantly interrupted, a 100% stimulation capture rate is still achieved. At a stimulation rate of 5 Hz or a period of 200 ms, the subsequent stimulation pulse is applied at 9.7 ms after the cardiac muscle reaches the contraction peak. However, the resulting capture rate decreases to 50%. For precise absolute refractory period (ARP) characterization, the stimulation frequency is increased up to 9 Hz corresponding to a period of 111 ms and the measured optical signal is shown in Fig. 6d. A 50% capture rate is achieved at this stimulation frequency, indicating an ARP of about 222 ms. This result is further verified by the corresponding extracellular potential recording (Fig. 6d). One evoked signal is observed for every two stimulation pulses, showing a 50% capture rate consistent with the optical signal. These measurements demonstrate that the dynamic optical recording modality can faithfully capture cardiomyocyte contractile responses, providing a dead-zone-free alternative to vulnerable electrical recording and sufficient timing resolution to measure functional parameters such as ARP.

**Fig. 7:**
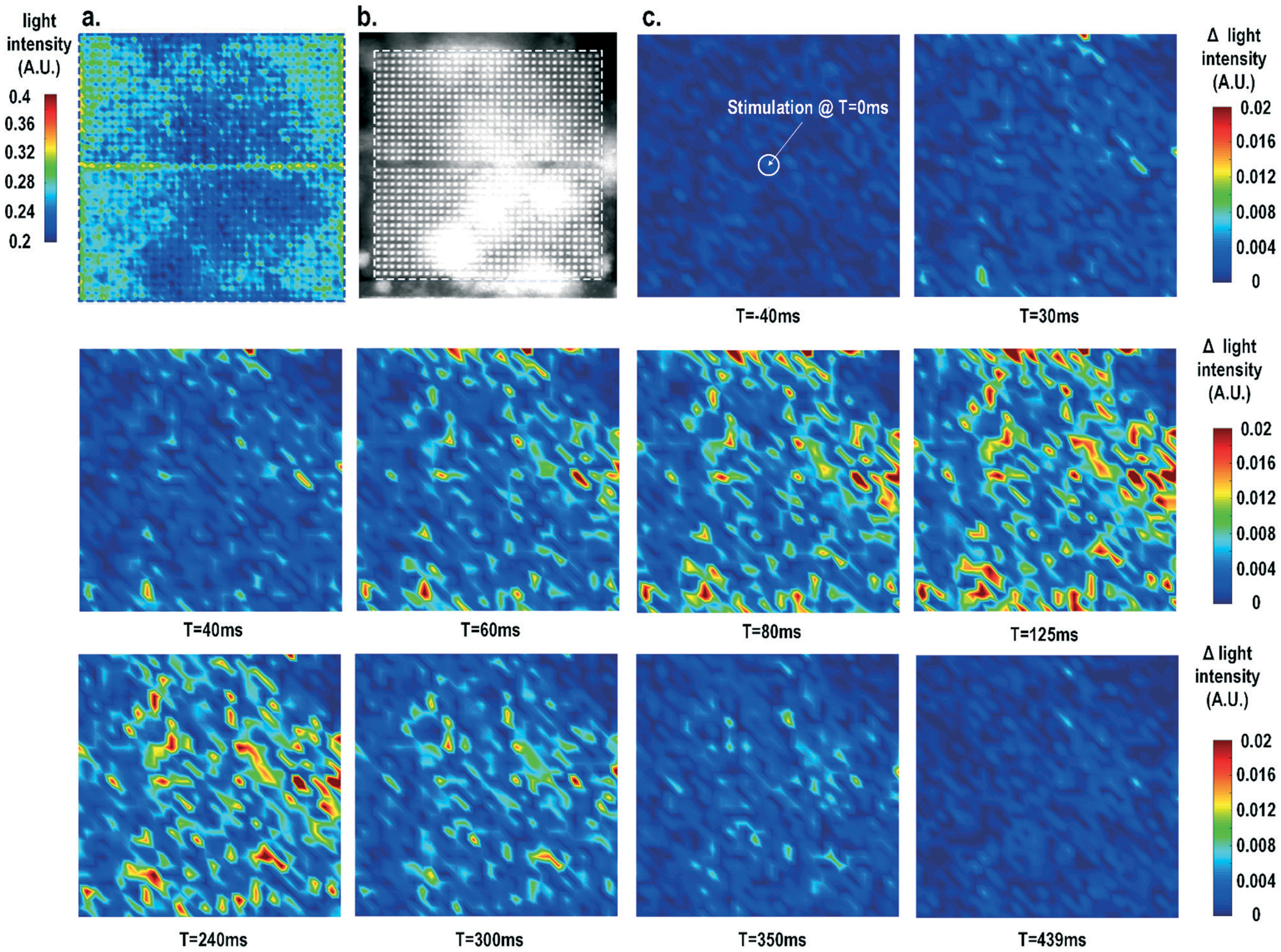
(a) The measured static optical opacity image and (b) the reference stereo-microscope image of on-chip cultured cardiomyocytes. (c) The measured time-lapse light intensity variations at 1024 pixels with stimulation (*t* = 0 ms) at the chip centre.

Furthermore, the real-time light intensities are sequentially measured at 1024 optical sensing pixels (activating 1 photodiode per pixel) throughout the CMOS chip with the electrical current stimulation at the centre of the chip at *t* = 0 ms (Fig. 7), showing the collective behaviour of the cardiomyocytes. Since our CMOS cellular interfacing array can precisely control the spatiotemporal properties of the stimulatory electrical signal and initiation of cardiac beating, the sequentially measured transient optical signals at 1024 pixels can be used to reconstruct the transient collective behaviour of the on-chip cultured rat cardiomyocytes throughout the entire sensing area upon stimulation. First, the measured static optical opacity image of on-chip cultured rat cardiomyocytes and the reference stereo-microscope image are shown in Fig. 7a and b. For the static optical opacity image, the entire 4096 optical sensing sites (1024 pixels with 4 photodiodes per pixel) are scanned to achieve a high resolution. The measured optical opacity image closely matches the reference stereo-microscope image and shows that the on-chip cultured cardiomyocytes achieve confluency and form an electrically coupled syncytium. Next, the collective behaviour of the on-chip cultured cardiomyocytes in response to the central electrical stimulation is recorded using sequentially measured real-time light intensities at all the 1024 optical sensing pixels (activating 1 photodiode per pixel). Note that there is no dead-zone for cell monitoring. The time-lapse light intensity changes (Δlight intensity) with respect to the reference light intensity are shown in Fig. 7c (1).

(1) ΔLight intensity(t)=Light intensity(t)−Light intensity(0)

A real-time synchronized collective cardiac muscle contraction and relaxation behaviour is observed, notably without any monitoring dead-zone. The on-chip cultured cardiomyocyte monolayer first contracts upon stimulation until it reaches its peak and then relaxes back to its baseline. Upon stimulation at the chip centre, the cardiomyocyte movement starts from the periphery and then propagates to the centre for both contraction and relaxation. This could be due to the stress build-up at the centre that strains the less constrained periphery first. Interestingly, this is distinct from action potential propagation that is initiated at the centre by stimulation and then propagates to the chip periphery.

### Multi-parametric feature extractions for holistic cellular characterization and drug screening

In this section, physiologically relevant parameters are defined and extracted based on parallel measured extracellular potentials and dynamic optical signals. This dual-modality framework will then be used to investigate the dose-dependent effects of isoproterenol, a known beta-adrenergic agonist, on the excitation–contraction dynamics of cardiomyocytes.

**Fig. 8:**
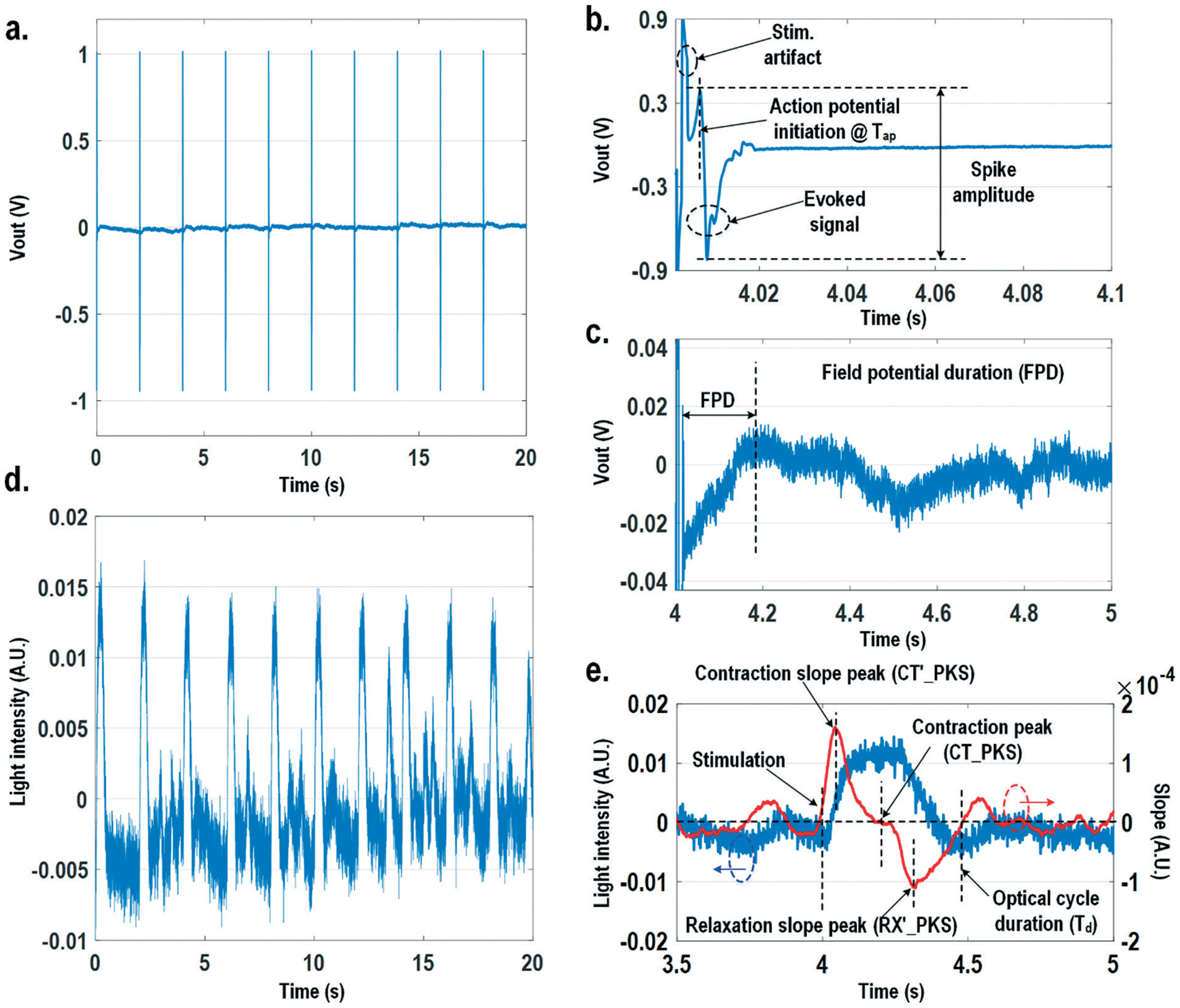
(a) The measured extracellular potentials with concurrent stimulation at 0.5 Hz. (b) The zoomed-in view of the measured extracellular action potential spike. (c) The zoomed-in view of the measured extracellular T-wave. (d) The measured optical signals with concurrent stimulation at 0.5 Hz. (e) The zoomed-in view of the measured optical signal with its time derivative.

Fig. 8 shows the measured extracellular potentials and dynamic optical signals upon stimulation at 0.5 Hz. In order to avoid saturation of the potential recording amplifier, the extracellular potential is recorded at a recording pixel 678 μm away from the stimulation site. To ensure biological correlations, dynamic optical recording is performed at the same recording pixel as the extracellular potential recording. The measured extracellular potentials are shown in Fig. 8a–c with concurrent stimulation. With a current stimulation pulse width of 1.2 ms and a 33 μA stimulation current amplitude, one evoked signal per stimulation pulse is observed for 10 continuous stimulations, achieving a 100% capture rate (Fig. 8a). The zoomed-in view of the measured extracellular potential signals (Fig. 8b and c) show stimulation artefacts (biphasic stimulation pulse), evoked action potential spikes, and extracellular T-wave. With this extracellular potential waveform, the action potential initiation time (*T*_ap_), action potential spike amplitude, and field potential duration (Fig. 8b and c) can be extracted, which are critical physiological parameters related to the propagation velocity, the amount of local ion flows, and the refractory periods of cardiomyocytes, respectively. In parallel, the measured optical signal is shown in Fig. 8d and e, showing a cyclic beating pattern for stimulation at 0.5 Hz. Fig. 8e shows the zoomed-in view of a single beating cycle together with its time derivative. Based on the time derivative waveform, the contraction slope peak time (CT′_PKS), contraction peak time (CT_PKS), relaxation slope peak time (RX′_PKS), and optical cycle duration (*T*_d_) can be extracted with respect to the stimulation time, which are closely related to intracellular calcium transients. In addition, since the extracellular potential signal and optical signal are measured on the same pixel with concurrent current stimulation, it achieves a good biological correlation. These two orthogonal domain signals and relevant parameters can be directly processed and extracted, such as the time delay between action potential initiation and muscle contraction initiation, which is particularly useful for drug screening to evaluate arrhythmogenic drug side effects.^66–69^

**Fig. 9:**
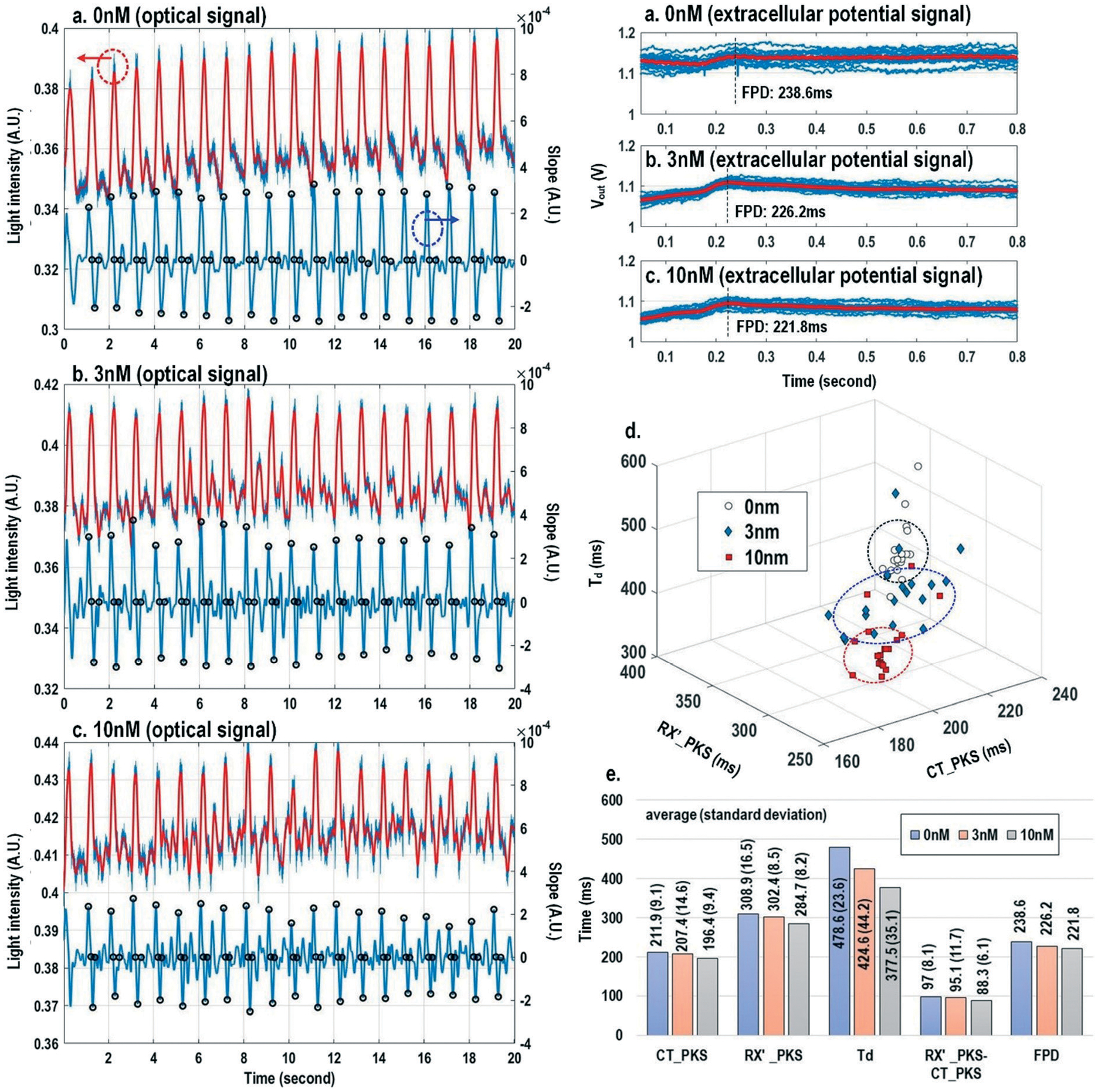
The measured extracellular potentials and optical signals with (a) 0 nM, (b) 3 nM, and (c) 10 nM isoproterenol concentration. (d) The 3-dimension and (e) hyper-dimension isoproterenol dose-dependent multi-parametric feature extractions.

Finally, the multi-parametric dose-dependent response of cardiomyocytes is studied using isoproterenol and feature extractions from their extracellular potentials and opto-mechanical signals are performed. Fig. 9a–c show the measured extracellular potentials and optical signals with increasing isoproterenol concentration. Since the intrinsic variability of the cell’s beating may interfere with parameter extractions, the on-chip biphasic current stimulator is used to regulate the beating rate at 1 Hz for this experiment. Fig. 9a shows the baseline extracellular potentials and optical signals without isoproterenol administration. The black circles in the time derivative optical signals in Fig. 9a–c indicate the optical time parameter extraction points, while the red lines in the extracellular potential signals are the averaged extracellular potential signals after stimulation. The number of measurements is defined as the measured cardiac beating counts for this experiment. For 0 nM, 3 nM, and 10 nM, the number of measurements (measured cardiac beating counts) are 19, 19, and 19, respectively. The measured field potential duration (FPD) is 238.6 ms; the measured contraction peak time (CT_PKS) is 211.9 ms with a standard deviation (*σ*) of 9.1 ms; the measured relaxation slope peak time (RX′_PKS) is 308.9 ms with a *σ* of 16.5 ms; the measured optical cycle duration (*T*_d_) is 478.6 ms with a *σ* of 23.6 ms. With an increased isoproterenol concentration of 3 nM (Fig. 9b), the mean values of these parameters decrease. For example, the measured FPD, CT_PKS, and RX′_PKS, and *T*_d_ decrease to 226.2 ms, 207.4 ms, 302.4 ms, and 424.6 ms, respectively. These parameters further decrease with the isoproterenol concentration of 10 nM (Fig. 9c). These measurement results are summarized in both 3-dimension and hyper-dimension plots. The results show that cardiac action potential cyclic processes are accelerated by beta adrenergic administration, which aligns well with the known mechanistic effects of isoproterenol.^70^

## Conclusions

In this paper, a CMOS quad-modality cellular interfacing array chip supporting multi-parametric and holistic cellular characterization for fully automated high-throughput drug screening and drug safety/toxicity assessments is presented. The CMOS quad-modality cellular interfacing array features extracellular potential recording, cellular impedance measurement, static/dynamic optical recording, and biphasic current stimulation. It contains 1024 multi-modal pixels with a 58 μm pixel pitch size in a total chip area of 2 mm by 3 mm, including all the front-end circuits and signal conditioning electronics.

With our CMOS quad-modality chip, label-free time-lapse cell transparency and cell-to-surface adhesion measurements based on on-chip cultured fibroblasts are demonstrated for cell growth and viability monitoring using two orthogonal modalities, *i.e*., the optical sensing modality and the impedance sensing modality. The measured optical opacity image shows the location and projected 2-D shape of the fibroblast aggregate as well as the opacity gradient within the fibroblast aggregate. The time-lapse optical opacity image tracks the cell opacity changes over time and captures that fibroblasts exhibit increasing optical transparency with time, due to desired cell proliferation and migration. In parallel, the measured cellular impedance monitors the cell-to-surface adhesion, and the time-lapse impedance measurement tracks the expansion of fibroblast adhesion on the CMOS chip surface over time. By comparing the optical opacity and impedance measurements, the cellular growth process and surface adhesion behaviour are monitored comprehensively.

Secondly, using on-chip cultured cardiomyocytes, simultaneous electrical current stimulation and cellular optical response monitoring are demonstrated without any monitoring dead-zone. The simultaneous stimulation and cellular response monitoring are essential to study electro-mechanically active cells such as cardiac cells. However, conventional electrical stimulation and extracellular potential recording systems suffer from a large dead-zone area around the stimulation site where the potential recording is prohibited due to the potential recording amplifier saturation by the stimulation artefacts. To address these challenges, simultaneous electrical current stimulation and optical cellular response monitoring are proposed. Upon stimulation, the synchronized cardiac muscle contraction and relaxation modulate the light transparency of cardiomyocytes and this light transparency modulation is captured by in-pixel photodiodes of the CMOS array. Simultaneous electrical current stimulation and optical recording on a single pixel at different stimulation rates are first demonstrated. Extracellular potential recording at a distant site is used for reference. Then, 1024 optical sensing pixels are sequentially scanned to measure the real-time collective behaviour of the on-chip cultured synchronized cardiomyocyte cluster upon stimulation. The measured transient optical signals at 1024 pixels show that upon stimulation at the chip centre, the cardiac muscle movements start from the chip periphery and then propagate to the centre both for contraction and relaxation, which could be due to the build-up of stress from the centre that strains the less constrained periphery first.

Finally, multi-parametric feature extractions are presented. Several critical physiological parameters such as the contraction slope peak time, the contraction peak time, the relaxation slope peak time, and the optical cycle duration are defined based on time derivatives of the recorded real-time optical signals of the cardiomyocytes, which are closely related to their intracellular calcium transients. Then, isoproterenol as an example drug is administrated to show the dose-dependent multi-parametric feature extractions. At different isoproterenol concentrations, the extracellular potentials and optical signals are measured at the same pixel to ensure a good biological correlation. The physiological parameters are extracted from extracellular potentials and optical signals and show that the action potential cyclic processes are consistently shortened with increased isoproterenol concentrations. Our CMOS quad-modality cellular interfacing array enables measurements of orthogonal physiological processes such as electrochemical and opto-mechanical responses on the same cellular sample with high biological correlation, supporting holistic cellular characterization and high throughput drug screening. As future work, new machine learning algorithms for multi-parametric feature extractions and classifications can be investigated and integrated into our CMOS quad-modality cellular interfacing array to facilitate drug screening/development and the discovery of dose-dependent patterns and relations among various physiological parameters.
